# Expression Profile and Potential Function of Circular RNAs in Peripheral Blood Mononuclear Cells in Male Patients With Primary Gout

**DOI:** 10.3389/fgene.2021.728091

**Published:** 2021-10-26

**Authors:** Fei Dai, Quan-Bo Zhang, Yi-Ping Tang, Yi-Xi He, Ting Yi, Yu-Feng Qing

**Affiliations:** ^1^ Research Center of Hyperuricemia and Gout, Affiliated Hospital of North Sichuan Medical College, North Sichuan Medical College, Nanchong, China; ^2^ Department of Rheumatology and Immunology, Affiliated Hospital of North Sichuan Medical College, North Sichuan Medical College, Nanchong, China; ^3^ Department of Geriatrics, Affiliated Hospital of North Sichuan Medical College, North Sichuan Medical College, Nanchong, China

**Keywords:** gout, circular RNA, microarray analysis, peripheral blood mononuclear cells, biomarker

## Abstract

Circular RNAs (circRNAs) are non-coding RNAs (ncRNAs) with a single-stranded covalently closed-loop structure, and their abnormal expression may participate in the pathogenesis of various human diseases. Currently, knowledge of circRNAs in gout is limited. In this case-control study, human circRNA microarrays were used to identify differentially expressed circRNAs in peripheral blood mononuclear cells (PBMCs) from patients with primary gout (*n* = 5) and healthy controls (HC; *n* = 3). Bioinformatics methods were used to analyze significantly different circRNAs (fold change >1.5, *p* < 0.05). In addition, four significantly differentially expressed circRNAs were selected for quantitative real-time polymerase chain reaction to detect expression levels in 90 gout patients and 60 HC. Subsequently, circRNA-miRNA-mRNA network was established to predict the function of circRNAs of interest. Microarray analysis indicated that 238 circRNAs were upregulated and 41 circRNAs were down-regulated in the gout group (fold change >1.5, *p* < 0.05). Bioinformatics analysis showed that differentially expressed circRNAs were involved in the pathogenesis of gout via various pathways. Moreover, the expression levels of hsa_circRNA_103657 and hsa_circRNA_000241 were significantly higher in the gout group than those in the HC group, and both correlated significantly with lipid metabolism parameters. Furthermore, the area under the curve of hsa_circRNA_103657 was 0.801 (95% confidence interval (CI): 0.730–0.871; *p* < 0.001). Our results provide novel insights into the pathogenesis of primary gout. Differentially expressed circRNAs were identified in the PBMCs of gout patients, and these differential circRNAs may play important roles in the development and progression of gout.

## Introduction

Gout is a common metabolic disease characterized by disturbances in purine metabolism and (or) decreased excretion of uric acid, which lead to increased uric acid in the blood and deposits of urate crystals in tissues (Dalbeth et al., 2016). The main manifestations of primary gout include hyperuricemia, acute arthritis, tophi, joint deformities, urinary tract stones, and kidney disease. Recent epidemiological studies have shown that the global prevalence and incidence of gout are increasing, with a prevalence of <1–6.8% and an incidence of 0.58–2.89 per 1,000 person years (Dehlin et al., 2020). However, the specific pathogenesis of gout remains unclear. Studies have reported that genetics, immunity, eating habits, and traumatic stress, are likely involved in the occurrence and development of gout (Dalbeth et al., 2016; Dalbeth et al., 2019; Clebak et al., 2020; Dehlin et al., 2020). Although elevated serum uric acid levels are considered an important risk factor for the development of gout, only approximately 10% of hyperuricemia patients suffer from gout (Zhang, 2021). The typical onset of joint symptoms and the discovery of urate crystals in the joint cavity are important indicators for the diagnosis of primary gout (Dalbeth et al., 2016), at which point urate crystals have been deposited into the joint cavity or caused joint deformities, which results in serious consequences for patients’ quality of life (Dalbeth et al., 2019). In addition, patients with gout often have comorbid diabetes, hypertension, hyperlipidemia, and cardiovascular and cerebrovascular diseases, which significantly threaten human health (Dalbeth et al., 2016; Dalbeth et al., 2019; Clebak et al., 2020; Dehlin et al., 2020). Therefore, clarifying the exact pathogenesis of gout and identifying biomarkers of primary gout is crucial to develop methods that enable early diagnosis and treatment of gout.

As an important element of epigenetics, non-coding RNA participates in the development of diseases by regulating gene expression and other functions; this is currently a research hotspot in the field of life sciences. Circular RNAs (circRNAs) are a new type of non-coding RNA molecule, which are composed of exons, introns, or fragments of the two, and are highly resistant to degradation because of their distinct circular structure (Zhou et al., 2020). Emerging evidence suggests that circRNAs are potential candidates for diagnostic biomarkers and therapeutic targets for a variety of diseases, including tumors (Wang J. et al., 2021), cardiovascular diseases (Ward et al., 2021), and nervous system diseases (Li M.-L. et al., 2021). However, at present, knowledge of circRNAs in gout remains limited. Therefore, in this study, microarray technology was used to screen and analyze the differential expression of circRNAs in peripheral blood mononuclear cells (PBMCs) between patients with primary gout and healthy controls (HC). We aimed to explore the role of circRNAs in gout, provide new insights into the pathogenesis of gout, and identify biomolecules for the diagnosis and treatment of gout from a genetic perspective.

## Materials and Methods

### Patients and Sample Collection

Male patients with primary gout and healthy volunteers were recruited from the Affiliated Hospital of North Sichuan Medical College from March 2020 to March 2021. All patients met the American College of Rheumatology/European League Against Rheumatism 2015 gout classification criteria (Neogi et al., 2015) and were subdivided into acute gout flare (AG) and intercritical gout (IG) groups according to their clinical manifestations. Specifically, gout patients with symptoms of joint swelling, heat, and pain during the previous 3 days were classified into the AG group, whereas those who had not experienced joint symptoms for at least 2 weeks and had normal inflammatory indicators, such as erythrocyte sedimentation rate (ESR), high-sensitivity C-reactive protein (CRP), and white blood cell (WBC) count, were classified into the IG group. During the same period, age- and sex-matched healthy volunteers with no hyperuricemia, metabolic syndrome, or other chronic diseases were recruited as the HC group from the Physical Examination Center of Affiliated Hospital of Sichuan North Medical University.

The study was conducted in two phases. Firstly, we selected six primary gout patients (three AG and three IG) and three HC and isolated total RNAs from their PBMCs for microarray analysis using a human circRNA microarray (Arraystar Inc., Rockville, MD, United States). Secondly, target circRNAs were detected in 90 primary gout patients (45 AG and 45 IG) and 60 HC using quantitative real-time polymerase chain reaction (qRT-PCR). Exclusion criteria for gout patients included 1) serious diseases of major organs, such as the heart and kidneys, 2) other serious conditions, such as malignant tumors and autoimmune diseases, 3) infection-related disorders, and 4) secondary gout due to other diseases.

The clinical data of all subjects including age, sex, body mass index, and laboratory indicators, such as serum uric acid (sUA) levels, blood glucose (GLU) levels, inflammation indicators, and lipid metabolism indicators were recorded. Inflammation indicators included ESR, CRP, WBC count, neutrophil granule count (GR), lymphocyte count (LY), and monocyte count, (Mo), and lipid metabolism indicators included plasma total cholesterol (TC), triglycerides (TG), high-density lipoprotein cholesterol (HDL), low-density lipoprotein cholesterol (LDL), and very-low-density lipoprotein (VLDL). All laboratory indicators were measured by the Clinical Laboratory Department of the Affiliated Hospital of North Sichuan Medical College. All participants provided written informed consent and patient data were analyzed anonymously. The study was approved by the Ethics Committee of the Affiliated Hospital of North Sichuan Medical College (Ethics approval number: 2019 ER(A)040) and was conducted following the ethical code of the Declaration of Helsinki of 1975.

### Sample Preparation

In each subject, 4 ml of fasting peripheral venous blood were collected in a heparin anticoagulation tube. PBMCs were isolated from blood samples by Ficoll-Hypaque density gradient centrifugation and stored at −80°C for subsequent analysis.

### RNA Extraction

The total RNA in PMBCs was extracted according to manufacturer instructions of the TRIzol reagent (Invitrogen, Grand Island, NY, United States). The integrity of RNA was evaluated by standard denaturing agarose gel electrophoresis, and the concentration and purity of RNA were measured by NanoDrop ND-1000.

### Circular RNAs Microarray

Samples of age- and sex-matched six gout patients (three AG and three IG) and three HC were selected for the microarray analysis. We used the Arraystar Human circRNA Array v2 chip (8 × 15 K) (Arraystar, Rockville, MD, United States), which contains 13,617 human circRNA probes. Sample preparation and microarray hybridization were performed according to Arraystar’s standard protocols (Agilent Technology). Briefly, 2000 ng of the total RNA were digested with rnase R (Epicentre, Illumina, Inc., Madison, WI, United States) to remove linear RNAs and enrich circRNAs. Then, the enriched circRNAs were amplified and transcribed into fluorescent cRNA using a random priming method (Arraystar Super RNA Labeling Kit; Arraystar). The labeled cRNAs were hybridized onto the Arraystar Human circRNA Array v2 (8 × 15 K, Arraystar). After washing the slides, the arrays were scanned using the Agilent Scanner G2505C (Agilent Technologies, Inc., Santa Clara, CA, United States).

### Data Analysis and Differentially Expressed Circular RNAs Identification

The Agilent Feature Extraction software (version 11.0.1.1) was used to analyze the acquired array images. Quantile normalization and subsequent data processing were performed using the R software (R Project for Statistical Computing, Vienna, Austria) limma package. Differentially expressed circRNAs that differed significantly between the gout group and the HC group were identified using scatter plot and volcano plot filtering (*p*-value calculated from an unpaired *t*-test; *p* < 0.05). Differentially expressed circRNAs between the two groups were identified using fold change filtering (fold change >1.5). Hierarchical clustering was performed to determine distinguishable circRNA expression patterns among samples.

### Quantitative real-time polymerase chain reaction Amplification

Four significantly more highly expressed circRNAs (hsa_circRNA_100632, hsa_circRNA_405646, hsa_circRNA_ 000241, and hsa_circRNA_103657) and two significantly more lowly expressed circRNAs (hsa_circRNA_104917, hsa_circRNA_001594) were selected from the microarray results for the qRT-PCR experiments.

According to the instructions of the reverse transcription reagent (TaKaRa, Japan), 2 µl (600 ng) of total RNA was reverse transcribed into cDNA. The PCR reaction was then performed using the LightCycle®96 PCR machine (Roche, Switzerland). The reaction system included TB Green Premix Ex Taq II (Takara Bio, Inc.): 5 μl, the forward primer: 0.1 µl (10 pmol/L), the reverse primer: 0.1 µl (10 pmol/L), cDNA: 1.0 µl, and ddH20: 3.8 µl. The thermocycling conditions were as follows: 95°C for 10 min, followed by 40 cycles of denaturation at 95°C for 10 s, and annealing/extension at 60°C for 1 min. All reactions were performed in triplicate. The expression of the housekeeping gene (β-actin) was used as the internal control to normalize the expression of each target gene. Relative quantification with the 2^−ΔΔCq^ method (Livak and Schmittgen, 2001) was used to evaluate the relative expression of circRNAs. The β-actin and the six circRNA primer sequences used for qRT-PCR (Shanghai Shenggong Bioengineering Co., Ltd., Shanghai, China) are listed in Table 1.

**TABLE 1:** Primer sequences used in the validation of circRNAs.

gene name	Forward primers sequence(5′–3′)	Reverse primers sequence(5′–3′)
β-actin	GAGCTACGAGCTGCCTGACG	GTAGTTTCGTGGATGCCACAG
hsa_circRNA_000241	GTGCGCTGCAATCCAGACAG	AGGACAAGCCGACTGCATTTG
hsa_circRNA_103657	ATGGACCCAAGAGTCAGCGG	TGCTTGGCCATTTCGGTTCC
hsa_circRNA_405646	ACGAGCTGGTTAGGAGGCAC	TACATCCACTCCACGGCACC
hsa_circRNA_100632	TTGTGACCTCCCTCTCCGTG	GCGGCGAATGCAGAGTTGAT
hsa_circRNA_104917	TTCAGCTCTGAGACCACCGC	TCATCTGCGAGAGGTCCCCT
hsa_circRNA_001594	GGTGCCTCCTGACTGCAGAA	ACTCGAGGGTGGAGGAGAGT

### Bioinformatics Analysis: Gene Ontology and Pathway Analysis

The gene ontology (GO) project provides a controlled vocabulary to describe gene and gene product attributes of any organism (http://www.geneontology.org) (Huntley et al., 2014). The ontology covers three domains: Biological Process (BP), Cellular Component (CC), and Molecular Function (MF). Fisher’s exact test in the Bioconductor’s top GO was used to establish whether there was more overlap between the differential genes and the GO annotation list than would be expected by chance. The *p*-value produced by the top GO denotes the significance of the GO items’ enrichment in the differential genes, where a lower *p*-value indicated a more significant GO item (*p* ≤ 0.05 was recommended).

Pathway analysis is a functional analysis that maps genes to Kyoto Encyclopedia of Genes and Genomes (KEGG) pathways (http://www.genome.jp/kegg/) (Kanehisa et al., 2014). The *p*-value (EASE-score, Fisher’s *p*-value, or hypergeometric *p-*value) denotes the significance of the pathway correlated to the conditions, where the lower the *p*-value, the more significant the pathway (*p* < 0.05 was considered statistically significant).

### Construction of Competitive Endogenous RNA Regulatory Network

CircRNA-miRNA interactions were predicted using Arraystar’s homemade miRNA target prediction software (Rockville, MD, United States) based on TargetScan (http://www.targetscan.org/vert_72/) (Enright et al., 2003) and miRanda (http://www.miranda.org/) (Pasquinelli, 2012). Then, the target genes of these miRNAs were predicted using the TargetScan, miRDB (http://mirdb.org/) (Chen and Wang, 2020), and mirtarbase (https://mirtarbase.cuhk.edu.cn/) (Huang et al., 2020) databases. Only the miRNA-mRNA predicted by these three databases were retained. Finally, according to the predicted relationship of circRNA-miRNA-mRNA, the ceRNA network was constructed using the Cytoscape software version 3.7.2 (http://www.cytoscape.org/index.html) (Shannon et al., 2003).

### Statistical Analysis

IBM SPSS Statistics 23.0 and GraphPad Prism 8.0 statistical software were used for analysis. Quantitative data with approximately normal distributions are described as means ± standard deviations, and data with non-normal distributions are described as medians (interquartile ranges). Statistical analysis of demographics, clinical, and laboratory indicators were performed using independent samples t-tests, Mann-Whitney U-tests, and one-way analyses of variance, followed by LSD posthoc tests. Fisher’s exact test was used for the GO enrichment and KEGG pathway analysis. Correlations were calculated using Spearman’s rank correlation test. The receiver operating characteristic (ROC) curve was used to evaluate the diagnostic efficacy of the candidate biomarker. A *p* < 0.05 was considered statistically significant.

## Results

### Circular RNAs Expression Profiles

Arraystar Human circRNA Array v2 (8 × 15 K) was used to detect and analyze circRNAs in the PBMCs of six gout patients (including three AG and three IG) and three HC. When data of nine subjects were analyzed and processed, the results of one IG case were found to be significantly biased. Through a retrospective review, the patient was found to have possible early rheumatoid arthritis at enrollment into the study, which was missed at that time of recruitment. Thus, the data of this case was excluded and the data of eight cases (three AG, two IG, and three HC) were retained for subsequent analyses. The demographic and clinical features of the eight subjects are summarized in Supplementary_Material S1. To ensure rigor, all subjects were reconfirmed that they met inclusion criteria.

**FIGURE 1:**
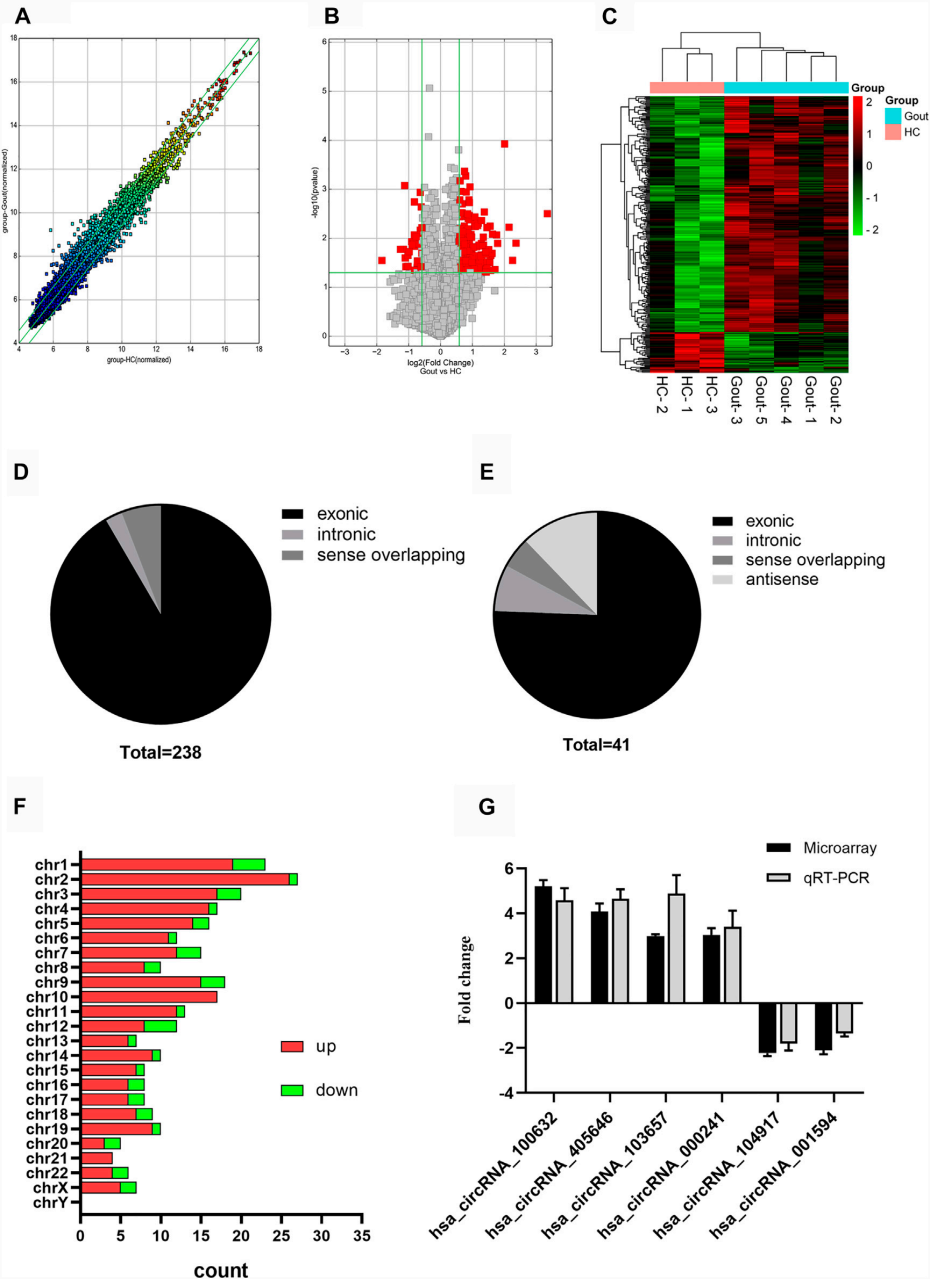
Expression profiles of circRNAs in gout patients compared with healthy controls. **(A)** Scatter plot demonstrating the heterogeneity of the expression of circRNAs in the gout and HC groups. The values of the *X* and *Y* axes represent the averaged normalized signal values of the groups (log2-scaled). The green line represents 1.5-fold changes. The expression of circRNAs above the top green line and below the bottom green line indicates changes by > 2-fold between the two groups. **(B)** Volcano plots visualizing differentially expressed circRNAs between the two groups. The vertical lines correspond to 1.5-fold up- and downregulated circRNA expression. The horizontal line represents a *p*-value of 0.05. The red point in the plot represents significantly differentially expressed circRNAs. **(C)** Clustered heatmap showing the relationships among the expression levels of samples. Expression values are represented by the color scale. The intensity increases from green (relatively low expression) to red (relatively high expression). Each column represents one sample, and each row represents a single circRNA. **(D)** Genomic region distribution of upregulated circRNAs. **(E)** Genomic region distribution of downregulated circRNAs. **(F)** Distribution of significantly dysregulated circRNAs in chromosomes. **(G)** Relative expression levels of the six circRNAs in five gout patients and three healthy control (HC) subjects. The Y-axis represents the ratio of the relative expression level of circRNAs in the gout group to that of the HC group. Gout: primary gout; HC: healthy controls.

We found 279 differentially expressed circRNAs (fold change >1.5 and *p* < 0.05) between gout and HC groups, of which 238 had higher and 41 had lower expression levels in the gout group than those in the HC group. Microarray data have been uploaded to the Gene Expression Omnibus database under the accession number GSE178825. The top 15 upregulated and top 15 downregulated circRNAs for the gout group are listed in Table 2. Scatter plots in Figure 1A show the heterogeneity of circRNA expression in the PBMCs of the gout and HC groups. The expression change of circRNAs above the top reference line and below the bottom reference line was >1.5-fold. Volcano plots (Figure 1B) were used to visualize the significantly differentially expressed circRNAs between the gout and HC groups. Hierarchical cluster analysis (Figure 1C) was used to show distinct circRNAs expression profiling between the gout and HC groups. We summarized the classification of significantly differentially expressed circRNAs (fold change >1.5, *p* < 0.05): 218 exons, 6 introns, 14 sense overlappings were included in the up-regulated circRNAs (Figure 1D); 31 exons, 3 introns, 2 sense overlappings, and 5 antisenses were contained in the downregulated circRNAs (Figure 1E). The transcripts of these significantly differentially expressed circRNAs were widely distributed in all chromosomes except the Y chromosome. The top two chromosomes that upregulated the distribution of circRNAs transcripts were chr1 (7.98%) and chr2 (10.92%), whereas in the downregulated circRNAs, chr1 and chr12 both accounted for 9.76%, and the other chromosomes were less than 8% (Figure 1F).

**TABLE 2:** The top 15 upregulated and 15 downregulated circRNAs in gout patients compared with healthy control subjects.

circRNA	Regulation	Fold Change	P-value	Alias	chrom	circRNA_type
hsa_circRNA_100914	up	10.2022483	0.003146809	hsa_circ_0023903	chr11	exonic
hsa_circRNA_100632	up	5.163444	0.012600554	hsa_circ_0018905	chr10	exonic
hsa_circRNA_091000	up	4.790979	0.028191498	hsa_circ_0091000	chrX	exonic
hsa_circRNA_403982	up	4.4366415	0.005949389	—	chr8	exonic
hsa_circRNA_405646	up	4.0272268	0.000118818	—	chr17	exonic
hsa_circRNA_000992	up	3.4618	0.012692591	hsa_circ_0000992	chr2	exonic
hsa_circRNA_007352	up	3.3827085	0.008540683	hsa_circ_0007352	chrX	exonic
hsa_circRNA_100332	up	3.2688998	0.043596282	hsa_circ_0014130	chr1	exonic
hsa_circRNA_100912	up	3.0983479	0.008225714	hsa_circ_0003695	chr11	exonic
hsa_circRNA_000241	up	3.0679421	0.020358477	hsa_circ_0000241	chr10	sense overlapping
hsa_circRNA_405746	up	2.9812781	0.022371618	—	chr19	exonic
hsa_circRNA_100925	up	2.9686745	0.04539817	hsa_circ_0023940	chr11	exonic
hsa_circRNA_103657	up	2.9388912	0.005663491	hsa_circ_0069977	chr4	exonic
hsa_circRNA_100923	up	2.9170916	0.031237871	hsa_circ_0023936	chr11	exonic
hsa_circRNA_103667	up	2.8591439	0.021060797	hsa_circ_0070039	chr4	exonic
hsa_circRNA_101287	down	3.5782171	0.028296353	hsa_circ_0008274	chr13	exonic
hsa_circRNA_101144	down	2.3865965	0.01677349	hsa_circ_0028196	chr12	exonic
hsa_circRNA_102339	down	2.233307	0.016725147	hsa_circ_0047351	chr18	exonic
hsa_circRNA_104917	down	2.1808263	0.000840297	hsa_circ_0088494	chr9	exonic
hsa_circRNA_407330	down	2.1543414	0.025214642	—	chrX	exonic
hsa_circRNA_001038	down	2.125022	0.037949687	hsa_circ_0000453	chr12	antisense
hsa_circRNA_007215	down	2.0316255	0.022281484	hsa_circ_0007215	chr4	sense overlapping
hsa_circRNA_047641	down	2.0271657	0.037410987	hsa_circ_0047641	chr18	exonic
hsa_circRNA_405498	down	1.973957	0.01876855	—	chr16	exonic
hsa_circRNA_100269	down	1.873869	0.011762548	hsa_circ_0013048	chr1	exonic
hsa_circRNA_102579	down	1.8471502	0.013598646	hsa_circ_0002311	chr19	exonic
hsa_circRNA_001594	down	1.846286	0.013111066	hsa_circ_0001343	chr3	antisense
hsa_circRNA_400100	down	1.7992879	0.012928107	hsa_circ_0092364	chr9	intronic
hsa_circRNA_066113	down	1.7671692	0.008504126	hsa_circ_0066113	chr3	exonic
hsa_circRNA_101501	down	1.7645955	0.001811216	hsa_circ_0034953	chr15	exonic

Four circRNAs (hsa_circRNA_100632, hsa_circRNA_405646, hsa_circRNA_000241, hsa_circRNA_103657) were randomly selected from the top 15 upregulated circRNAs, and two circRNAs (hsa_circRNA_104917, hsa_circRNA_001594) were randomly selected from the top 15 downregulated circRNAs for further analysis. The expression levels of six circRNAs in eight samples previously used for microarray detection were analyzed by qRT-PCR to verify the reliability of the microarray results. In the microarray analysis, the fold changes of the normalized levels of the six circRNAs between the gout and HC groups were 5.16, 4.03, 3.08, 2.94, 2.18, and 1.85. In the qRT-PCR analysis, the fold changes in the expression levels of the six circRNAs between the two groups were 4.59, 4.65, 3.39, 4.82, 1.80, and 1.36, respectively. Therefore, the qRT-PCR results were consistent with the trend of the microarray data (Figure 1G).

### Gene Ontology and Encyclopedia of Genes and Genomes Pathway Analyses of Differentially Expressed Circular RNAs

In this study, GO and KEGG pathway analyses were performed for the parental genes with significant differential expression of circRNAs in the microarray expression profile to explore the potential function and possible biological pathways of circRNAs in the PBMCs of gout patients. In the GO analysis, the histograms represent the top 10 items of enrichment scores in the upregulated (Supplementary Material S2A) and downregulated genes (Supplementary Material S2B). Differential genes were primarily related to the “cAMP biosynthetic process,” “enzyme binding,” “adenylate cyclase activity,” and the “nuclear ubiquitin ligase complex.” In the KEGG pathway analysis, the numbers of pathway items that differentially expressed higher and lower circRNAs in the gout group were 34 and 43, respectively. The KEGG pathway analysis showed the circRNAs more highly expressed in the gout group than those in the HC group mainly involved “mitophagy,” “ubiquitin-mediated proteolysis,” and the “FoxO signaling pathway” (Supplementary Material S2C; top 10 pathways), whereas circRNAs with lower expression levels in the gout group were significantly involved in “platelet activation,” the “apelin signaling pathway,” and the “cGMP-PKG signaling pathway” (Supplementary Material S2D; top 10 pathways).

### Expression Levels of the Four Circular RNAs in Gout Patients and Healthy Controls Subjects

**FIGURE 2:**
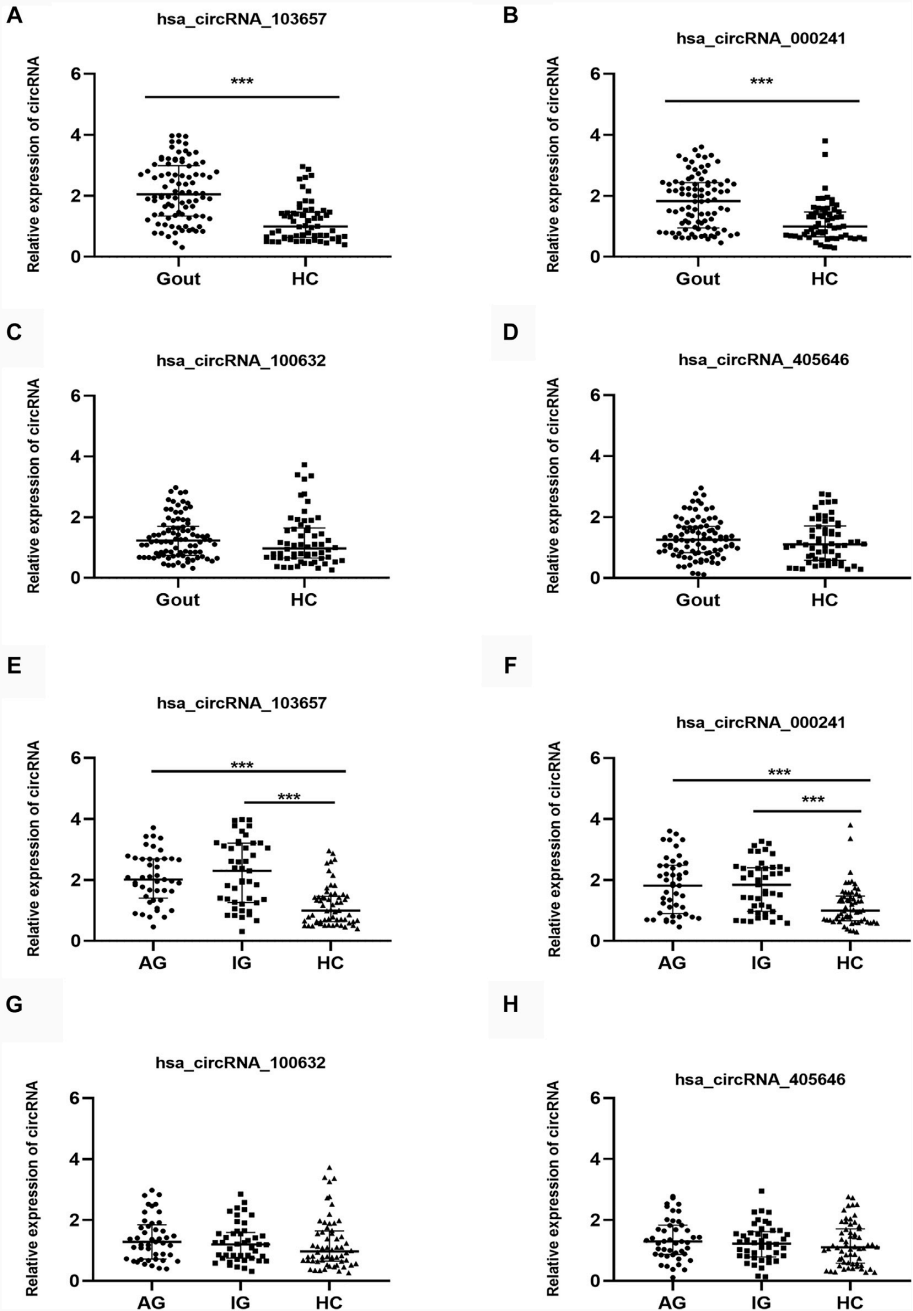
Expression levels of the four circRNAs in gout patients and healthy control subjects. **(A, B, C, D)** Relative expression levels of the four circRNAs in 90 gout patients and 60 healthy control (HC) subjects. **(E, F, G, H)** Relative expression levels of the four circRNAs in 45 acute gout glare (AG) patients, 45 intercritical gout (IG) patients, and 60 HC. Gout: primary gout, including acute gout flare and intercritical gout; AG: patients with acute gout flare; IG: patients with intercritical gout; HC: healthy controls; ***p < 0.001.

In the verification phase, the expression levels of the four upregulated circRNAs were significantly different between the gout and HC groups. Therefore, we detected the expression levels of the four upregulated circRNAs in 150 samples (45 AG, 45 IG, and 60 HC) by qRT-PCR to identify the most suitable clinical biomarkers and determine the role of circRNAs in gout. The demographic and clinical features of all the subjects are summarized in Table 3. As shown in Figure 2, the expression levels of hsa_circRNA_103657 and hsa_circRNA_000241 in the gout group were significantly higher than those in the HC group (*p* < 0.05; Figures 2A,B). The expression levels of hsa_circRNA_100632 and hsa_circRNA_405646 were not statistically different between the two groups (*p* > 0.05; Figures 2C,D).

**TABLE 3:** Clinical and laboratory data of subjects studied.

	Gout group (*n*=90)	AG group (*n*=45)	IG group (*n*=45)	HC group (*n*=60)
Age (years)	40.90 ± 12.42	41.31 ± 12.76	40.49 ± 12.20	42.63 ± 10.94
Gender F/M	0/90	0/45	0/45	0/60
Disease duration, median (range) (months)	37.50 (23.75–49.25)	24 (2.00–66.00)	39 (31.50–47.50)	—
BMI (kg/m^2^) ( $\bar{x}$ ±SD)	25.15 ± 3.47^a^	24.76 ± 3.63^a^	25.54 ± 3.29^a^	22.23 ± 2.06
CRP (mg/L) ( $\bar{x}$ ±SD)	15.06 ± 29.10	27.45 ± 37.31^b^	2.67 ± 2.57	—
ESR (mm/h) ( $\bar{x}$ ±SD)	15.90 ± 20.35	24.93 ± 25.49^b^	6.87 ± 4.58	—
WBC (×10^9^/L) ( $\bar{x}$ ±SD)	7.92 ± 2.79^a^	9.02 ± 3.08^ab^	6.83 ± 1.96^a^	5.54 ± 1.24
GR (×10^9^/L) ( $\bar{x}$ ±SD)	5.24 ± 2.48^a^	6.23 ± 2.75^ab^	4.24 ± 1.69^a^	3.38 ± 0.89
LY (×10^9^/L) ( $\bar{x}$ ±SD)	1.99 ± 0.68	2.00 ± 0.80^b^	1.98 ± 0.53	1.76 ± 0.59
Mo (×10^9^/L) ( $\bar{x}$ ±SD)	0.48 ± 0.21^a^	0.56 ± 0.23^a^	0.40 ± 0.16^a^	0.32 ± 0.08
sUA (μmol/L) ( $\bar{x}$ ±SD)	463.96 ± 142.45^a^	477.71 ± 140.47^a^	450.21 ± 144.65^a^	363.45 ± 51.66
GLU (mmol/L) ( $\bar{x}$ ±SD)	5.72 ± 1.12^a^	5.68 ± 1.07^a^	5.76 ± 1.17^a^	4.90 ± 0.41
TG (mmol/L) ( $\bar{x}$ ±SD)	2.43 ± 1.78^a^	2.18 ± 1.41^ab^	2.68 ± 2.09^a^	1.18 ± 0.33
TC (mmol/L) ( $\bar{x}$ ±SD)	4.83 ± 0.85^a^	4.61 ± 0.79	5.05 ± 0.87^a^	4.50 ± 0.64
HDL (mmol/L) ( $\bar{x}$ ±SD)	1.12 ± 0.26^a^	1.11 ± 0.26	1.13 ± 0.26	1.18 ± 0.20
LDLC (mmol/L) ( $\bar{x}$ ±SD)	2.78 ± 0.62	2.68 ± 0.59	2.89 ± 0.65	2.61 ± 0.58
VLDL (mmol/L) ( $\bar{x}$ ±SD)	0.95 ± 0.42^a^	0.89 ± 0.34^a^	1.02 ± 0.47^a^	0.64 ± 0.15

Gout: primary gout, including acute gout flare and intercritical gout; AG: patients with acute gout flare; IG: patients with intercritical gout; HC: healthy controls ; $\bar{x}$ ±SD: mean ± standard deviation; BMI：Body Mass Index; CRP: reactive protein; ESR: erythrocyte sedimentation rate; WBC: white blood cell counts; GR: neutrophile granulocytecounts; LY: lymphocyte counts; Mo: monocyte counts; sUA：serum uric acid; GLU: serum glucose; TG: triglycerides; TC: Total Cholesterol; HDL: high-density lipoprotein; LDL: low-density lipoprotein; VLDL: very low-density lipoprotein.

One-way ANOVA, T test or Mann Whitney test. p-value < 0.05 was considered to denote statistical significance

a in comparison with HC group

b in comparison with IG group.

In addition, the expression levels of the four circRNAs in the AG, IG, and HC groups are shown separately. The expression levels of hsa_circRNA_103657 and hsa_circRNA_000241 in the AG and IG groups were significantly higher than those in the HC group (*p* < 0.05) but did not differ significantly between the AG and IG groups (Figures 2E,F). The expression levels of hsa_circRNA_100632 and hsa_circRNA_405646 among the three groups were not statistically significant (Figures 2G,H).

### Prediction and Annotation of hsa_Circular RNAs_103657 and hsa_circRNA_000241 Circular RNAs Mechanism

**FIGURE 3:**
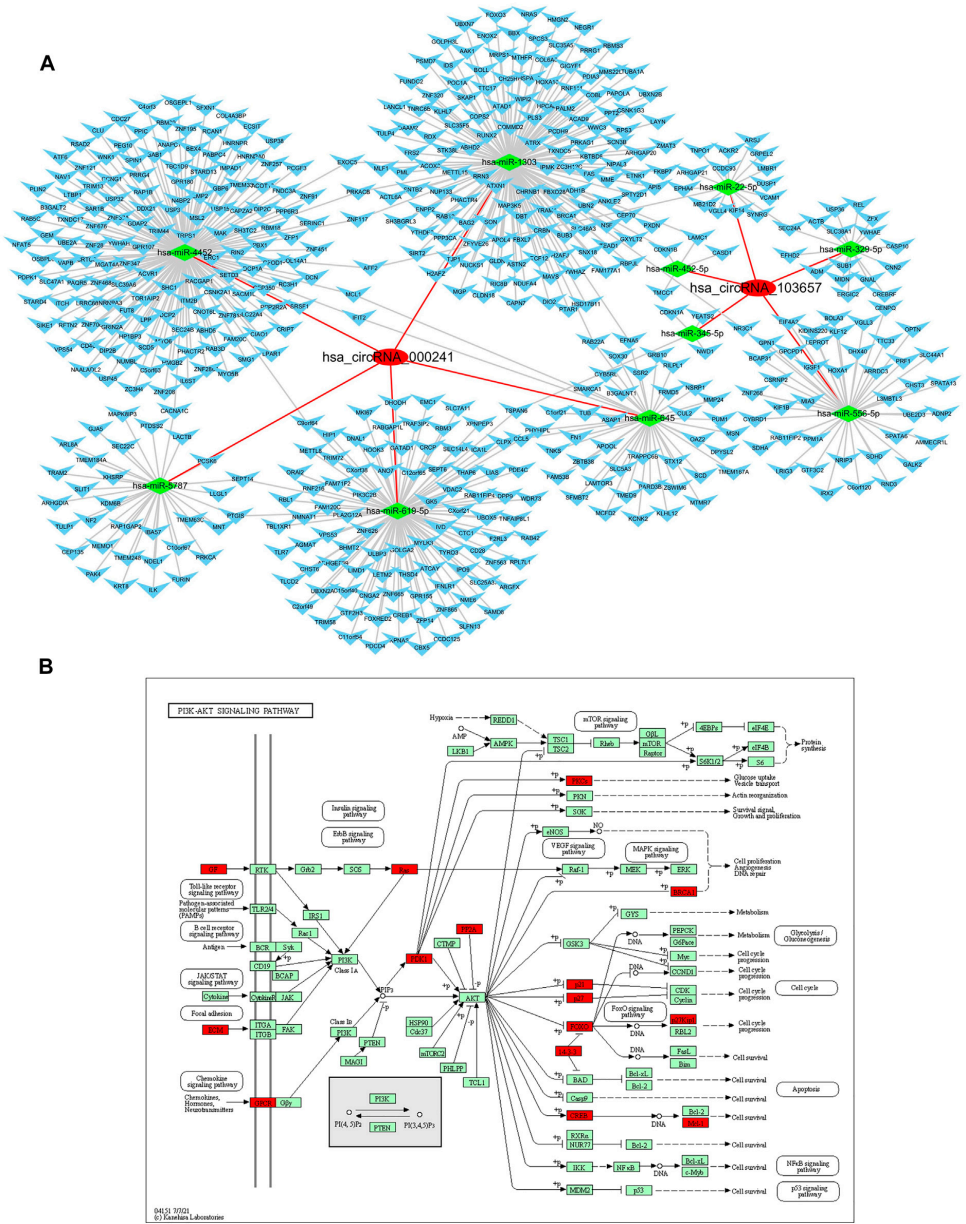
Prediction and annotation of hsa_circRNA_103657 and hsa_circRNA_000241 ceRNA mechanism. **(A)** Construction of the circRNA-miRNA-mRNA network: the network of ceRNA includes 2 circRNAs, 10 miRNAs, and 525 mRNAs. **(B)** Schematic diagram of the gene category of the PI3K-Akt signaling pathway, which is the top enriched term in the KEGG pathway analysis. Image from the Kyoto Encyclopedia of Genes and Genomes (KEGG) resource (http://www.genome.jp/kegg/).

Since some circRNAs can perform the function of “miRNA sponge” by efficiently binding and inhibiting miRNA transcription, which would further influence downstream mRNA expression and finally involved in various diseases, so we predicted the downstream miRNAs of severely dysregulated hsa_circRNA_103657 and hsa_circRNA_000241. Ranked by mirSVRscore, the top-5 miRNAs related to hsa_circRNA_103657 were hsa-miR-329-5p, hsa-miR-556-5p, hsa-miR-452-5p, hsa-miR-345-5p, hsa-miR-22-5p, and the top-5 miRNAs related to hsa_circRNA_000,241 were hsa-miR-1303, hsa-miR-619-5p,hsa-miR-645,hsa-miR-4452,hsa-miR-5787. MiRNA-targeted mRNAs were predicted by TargetScan, miRanda and mirtarbase. Finally, the predicted hsa_circRNA_103657 and hsa_circRNA_000241 targeted circRNA-miRNA-mRNA network was established based on sequence-pairing prediction, and a total of 10 miRNAs and 525 mRNAs were predicted to have an interaction with these two circRNAs (Figure 3A). Among them, hsa_circRNA_000,241-miRNAs exhibited a relatively large interaction network, especially with hsa-miR-1303, hsa-miR-619-5p, hsa-miR-4452. To gain further insights into the functions of these two circRNAs, all predicted mRNAs were subjected to GO and KEGG enrichment analysis. In the ceRNA regulatory network, GO analysis showed that mRNAs of 525 were mainly concentrated in “endomembrane system organization,” “COPII-coated vesicle budding” (categorized as BPs), “spindle,” “COPII vesicle coat” (categorized as CCs), “alpha-tubulin binding,” “kinesin binding” (categorized as MFs) (Supplementary Material S3). KEGG pathway analysis results show mainly enrichment in the ‘PI3K-Akt signaling pathway’ (Figure 3B) (Supplementary Material S4).

### Association Between hsa_Circular RNAs_103657 and hsa_Circular RNAs_000241 Expression Levels and Laboratory Data in Patients With Gout

**FIGURE 4:**
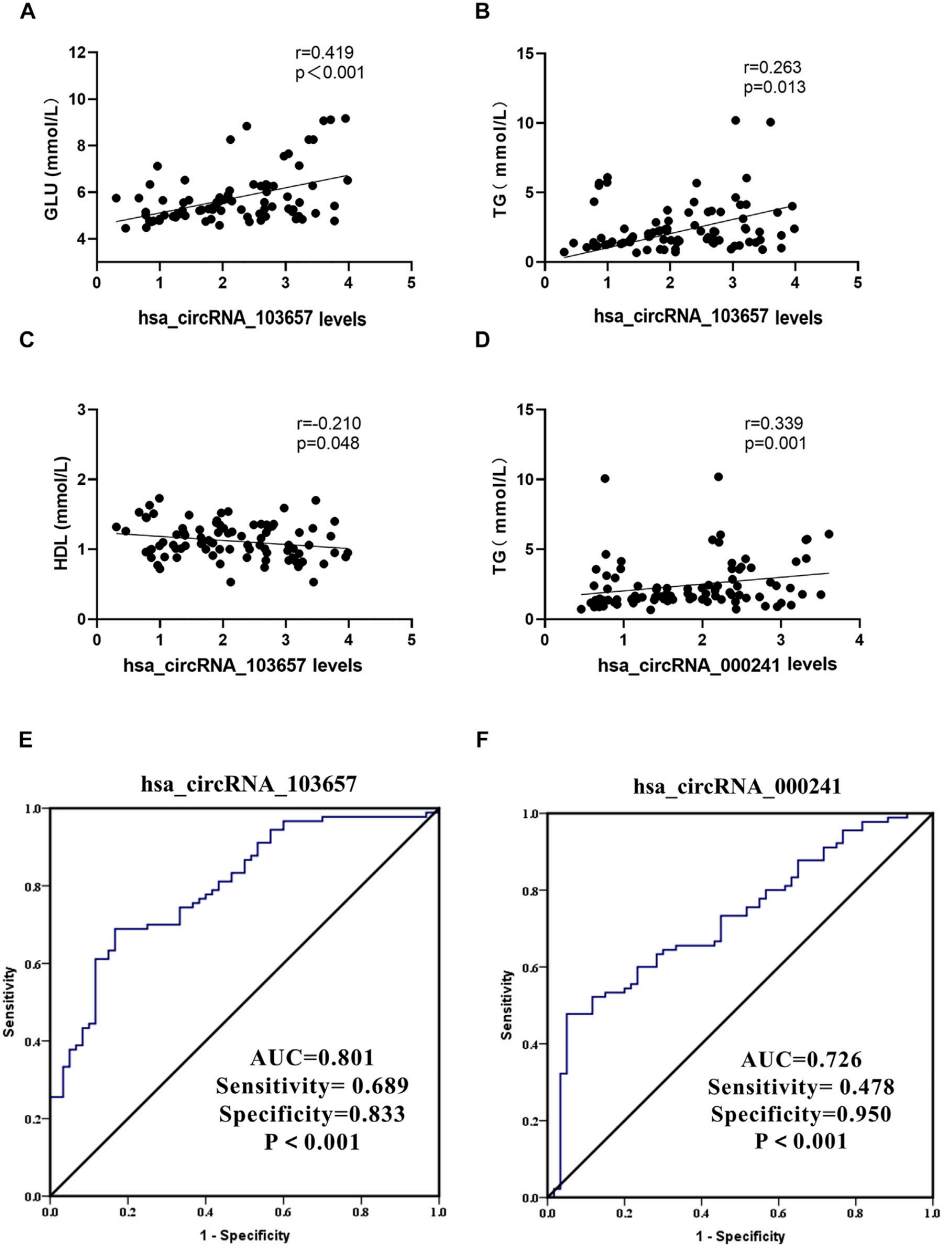
Clinical significance of hsa_circRNA_103657 and hsa_circRNA_000241 in primary gout **(A,B,C,D)** Correlations between the expression levels of circRNAs (hsa_circRNA_103657 and hsa_circRNA_000241) and laboratory data of gout, analyzed using Spearman’s coefficient. **(E, F)** Receiver operating characteristic (ROC) curve for the analysis of the diagnostic value of hsa_circRNA_103657 and hsa_circRNA_000241 for primary gout. AUC: area under the curve.

In the gout group, we assessed correlations between the expression levels of circRNAs (hsa_circRNA_103657 and hsa_circRNA_000241) and plasma lipid, sUA, and GLU levels. Additionally, we evaluated correlations between the expression levels of circRNAs (hsa_circRNA_103657 and hsa_circRNA_000241) and inflammation indicators in the AG group. Results suggested that the expression level of hsa_circRNA_103657 was positively correlated with GLU and TG (R = 0.419, *p* < 0.001; R = 0.263, *p* = 0.013, respectively; Figures 4A,B), negatively correlated with HDL (R = −0.210, *p* = 0.048; Figure 4C), and not correlated with sUA, TC, LDL, or VLDL. The expression level of hsa_circRNA_000241 was positively correlated with TG (R = 0.339, *p* = 0.001; Figure 4D) but not correlated with sUA, GLU, TC, HDL, LDL, or VLDL. There were no significant correlations between the expression levels of circRNAs (hsa_circRNA_103657 and hsa_circRNA_000241) and inflammation indicators in the AG group.

### Diagnostic Value of hsa_Circular RNAs_103,657 and hsa_Circular RNAs_000241 for Gout

ROC curves were generated to determine the diagnostic value of hsa_circRNA_103657 and hsa_circRNA_000241 in PBMCs for the diagnosis of primary gout (Figures 3E,F). The areas under the curve (AUC) of hsa_circRNA_103657 and hsa_circRNA_000241 for diagnosing primary gout were 0.801 (95% confidence interval (CI), 0.730–0.871; *p* < 0.001; sensitivity = 68.9%, specificity = 83.3%) and 0.726 (95% CI, 0.646–0.807; *p* < 0.001; sensitivity = 47.8%, specificity = 95.0%), respectively.

## Discussion

CircRNAs can act as miRNA sponges, interact with RNA binding proteins, affect protein translation, regulate protein recruitment, and modulate protein assembly (Zhou et al., 2020). Therefore, the multi-functionality of circRNAs makes them ideal for researching and predicting disease. In recent years, increasing numbers of studies have shown that the abnormal expression of circRNAs is closely related to the development of a variety of rheumatisms, such as systemic lupus erythematosus (Wang X. et al., 2021), rheumatoid arthritis (Cai et al., 2021), and osteoarthritis (Liu et al., 2021), etc., However, the role of circRNAs in gout is not well understood. In this study, genome-wide microarrays were used to analyze the circRNA expression profile in the PBMCs of gout patients. Compared with the HC group, the gout group had 238 upregulated and 41 downregulated circRNAs. This indicated that there were abnormally expressed circRNAs in the PBMCs of patients with gout, and these abnormally expressed circRNAs play a role in the pathogenesis of gout. According to the genomic regions, the general characteristics of differential circRNAs were further summarized, and most differentially expressed circRNAs were derived from exons. This indicated that circRNAs generate additional transcripts from a gene locus, which is consistent with recent research (Kelly et al., 2015) demonstrating that most circRNAs originate from the exons of protein-coding genes. These significantly different circRNAs were widely distributed in most chromosomes, including the X chromosome, which may be related to the biological mechanism underlying gout. QRT-PCR was used to detect the expression levels of six circRNAs in the PBMCs of eight subjects used in the initial microarray, and their expression levels were entirely consistent with the subsequent microarray results, which confirmed the reliability of the gene microarray data.

The analysis of the GO and KEGG pathways further explored the biological functions and potential mechanisms of the differentially expressed circRNAs in gout. Our results revealed that the biological functions of high- and low-expressed genes in gout differed significantly. Previous studies have confirmed that Toll-like receptor and NOD-like receptor signal transduction pathways are related to the pathogenesis of gout inflammation (Dalbeth et al., 2016; So and Martinon, 2017). Interestingly, our KEGG analysis found that the different circRNAs in the gout group mainly participated in “FoxO signaling pathway,” “apelin signaling pathway,” and “cGMP-PKG signaling pathway,” except for the Toll-like receptor and NOD-like receptor signaling pathways. These data indicated that circRNAs may be involved in the pathogenesis of gout via various signaling pathways.

The cause of gout is complex. Numerous studies (Dalbeth et al., 2016; So and Martinon, 2017; Dalbeth et al., 2019; Clebak et al., 2020; Dehlin et al., 2020; Zhang, 2021) have highlighted that the environment, diet, genetics, immunity, and other aspects participate in the development of the disease in varying degrees, and gout is more prevalent in the male population. We detected the expression levels of hsa_circRNA_100632, hsa_circRNA_405646, hsa_circRNA_103,657, and hsa_circRNA_000,241 in 150 male subjects (45 AG, 45 IG, and 60 HC) using qRT-PCR and confirmed that hsa_circRNA_103657 and hsa_circRNA_000241 were significantly upregulated in the PBMCs of gout patients. These circRNAs have not been reported to date. To gain further insight, we constructed a circRNA-miRNA-mRNA network based on hsa_circRNA_103657 and hsa_circRNA_000241, and conducted bioinformatics analysis on its downstream targets. The 10 predicted miRNAs have been reported in multiple diseases, especially hsa-miR-1303. It has been reported that hsa-miR-1303 plays an important role in osteosarcoma progression (Han et al., 2021), non-small cell lung cancer (Chen et al., 2020), breast cancer (Liang et al., 2020), Neuroblastoma (Li et al., 2016) and other diseases. KEGG enrichment analysis also pointed out effective signaling pathways, such as the PI3K-Akt signaling pathway, 18 predicted mRNAs participated in this pathway in our research. The dysfunction of PI3K-Akt signaling pathway was related to a variety of pathological conditions, including metabolic diseases (Zhang et al., 2021a), autoimmune inflammatory diseases (Song et al., 2019), cancers (Hoxhaj and Manning, 2020), and neurological diseases (Fu et al., 2020). There are also reports suggesting that the imbalance of the PI3K-Akt signaling pathway can affect uric acid metabolism (Zhang et al., 2021b) or gout inflammation (Paré et al., 2021). Our results indirectly indicate that the dysregulated hsa_circRNA_103657 and hsa_circRNA_000241 may participate in the pathogenesis of gout by affecting this pathway. This provides a theoretical basis for us to further explore the potential pathogenesis of circRNA-miRNA-mRNA regulatory network in gout.

Epidemiological investigations have shown that gout is closely related to hyperuricemia, hyperlipidemia, and hyperglycemia (Dalbeth et al., 2019). Moreover, elevated sUA levels are considered an important risk factor for gout, and hyperuricemia is one of the independent risk factors for metabolic diseases such as hypertension, diabetes, and coronary heart disease (Dalbeth et al., 2016; Zhang, 2021). Based on these findings, we analyzed the correlations between sUA, lipid, and glucose metabolism and the expression level of circRNAs and revealed that the expression level of hsa_circRNA_000241 was positively correlated with TG, and the expression of hsa_circRNA_103657 was positively correlated with TG and GLU and negatively correlated with HDL. This suggests that hsa_circRNA_000241 and hsa_circRNA_103657 are involved in the regulation of lipid or glucose metabolism in patients with gout and that increased expression levels are dangerous for patients with gout.

Gout is a metabolic disease that seriously affects people’s health. Although most patients with AG also have hyperuricemia, some gout patients exhibit sUA levels in the normal range. A retrospective cohort study in South Korea showed that sUA levels did not increase during an acute flare in approximately 40% of gout patients (Lee et al., 2020). Currently, the gold standard diagnostic test for gout is the identification of monosodium urate (MSU) crystals using polarized light microscopy in synovial fluid cells or a tophus (Neogi et al., 2015). However, because of the invasive nature of the operation, its clinical application has certain limitations, especially in gout patients without hyperuricemia, in whom the detection rate of MSU is extremely low. Therefore, it is vital to identify more effective diagnostic markers and therapeutic targets to prevent malignant development of the disease.

The ideal biomarker should possess the following characteristics: high specificity, high sensitivity, non-invasive, convenient, cheap, and reproducible. Because of their unique structure, high stability, and specific expression, circRNAs are being increasingly recognized as new diagnostic markers of diseases in recent years (Zhou et al., 2020; Wang J. et al., 2021; Ward et al., 2021). For example, circ-PSD3 in tissue samples may be a potential diagnostic biomarker or molecular therapy target for papillary thyroid carcinoma (Li Z. et al., 2021). Moreover, circFKBP8 and circMBNL1 are abnormally expressed in peripheral blood and may be potential diagnostic biomarkers for major depressive disorder (Shi et al., 2021). Luo et al. (Luo et al., 2021) have revealed that hsa_circ_0082688 and hsa_circ_0082689 in whole blood could be used as diagnostic biomarkers for systemic lupus erythematosus. However, there have not been any reports regarding the use of circRNAs as biomarkers for gout. In this study, the expression levels of hsa_circRNA_103657 and hsa_circRNA_000241 in the gout group were significantly higher than those in the HC group. As known to all, the ROC curve is generated using an analysis method that reflects the sensitivity and specificity of disease diagnosis indicators (Mandrekar, 2010). The AUC indicates the diagnostic efficacy of the indicator. The value of AUC ranges from 0.5 to 1.0, and values close to 1.0 indicate better diagnostic efficacy. The ROC analysis showed that the AUC of hsa_circRNA_103657 was more than 0.8, which indicated that it could distinguish gout patients from healthy individuals. Thus, hsa_circRNA_103657 may be offered as a potential diagnostic biomarker for gout. However, further validation is necessary to establish this biomarker for clinical use.

There are several limitations to the present study. First is the relatively small sample size and the inclusion of only male subjects. The findings need to be confirmed in large-scale studies conducted in populations with different ethnicities and from other regions. Second, to determine whether hsa_circRNA_103657 can be used as a diagnostic biomarker, it must be evaluated for its ability to accurately differentiate gout from other similar diseases. Cases with other conditions should be included in future studies to provide further evidence supporting hsa_circRNA_103657 as a diagnostic biomarker for gout. Third, we could only speculate that hsa_circRNA_103657 is involved in lipid and sugar metabolism in primary gout; the specific pathogenesis of hsa_circRNA_103657 in gout remains uncertain. Therefore, functional experimental studies are required to establish the causal relationship between aberrantly expressed hsa_circRNA_103657 and gout.

In conclusion, our study is the first to measure circRNA expression in the PBMCs of gout patients and HC using microarray technology and qRT-PCR. Our findings enhance our understanding of the role played by circRNAs in primary gout, especially hsa_circRNA_103657 and hsa_circRNA_000241, which has potential functional and clinical significance in gout. However, the molecular mechanism and specific functions of these circRNAs in gout require further study.

## Data Availability

We have uploaded the raw data to GEO accession number GSE178825: https://www.ncbi.nlm.nih.gov/geo/query/acc.cgi?acc=GSE178825).
