# Supplementary material for: Expression Profile and Potential Function of Circular RNAs in Peripheral Blood Mononuclear Cells in Male Patients With Primary Gout
Source: Front Genet. 2021 Oct 26;12:728091. doi: 10.3389/fgene.2021.728091 (PMC8576385; doi:10.3389/fgene.2021.728091)
Supplement: Supplementary file 1 [file DataSheet2.pdf]

A

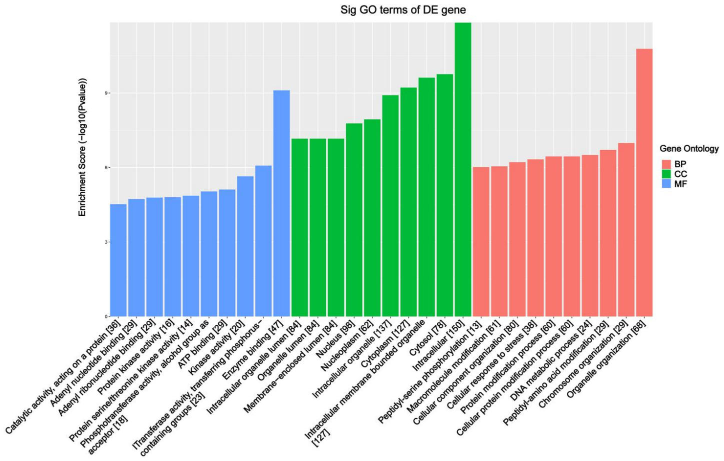

B

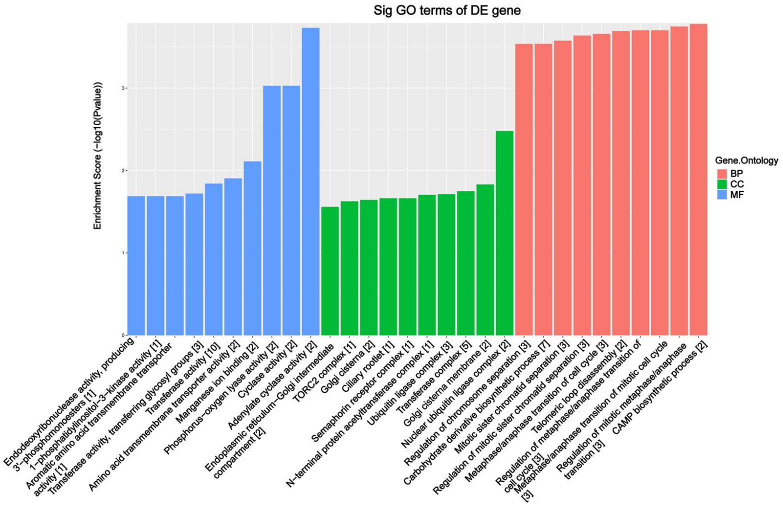

C

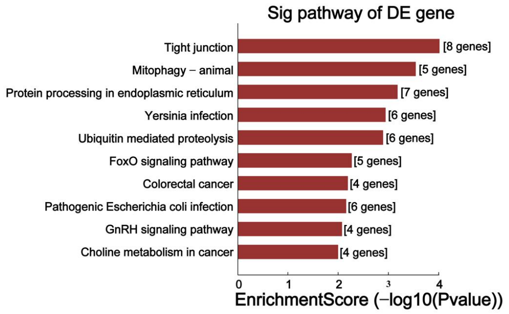

D

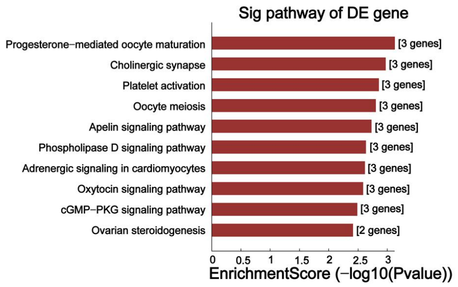

Supplementary_Materia S2 Bioinformatics analysis.

(A, B) Gene Ontology(GO) analysis enriches the top 10 items in the molecular function, cell composition, and biological process of upregulated and downregulated circRNAs.

(C, D) Kyoto encyclopedia of genes and genomes (KEGG) analysis enriched the top 10 items of upregulated and downregulated circRNAs.
