# Supplementary material for: Expression Profile and Potential Function of Circular RNAs in Peripheral Blood Mononuclear Cells in Male Patients With Primary Gout
Source: Front Genet. 2021 Oct 26;12:728091. doi: 10.3389/fgene.2021.728091 (PMC8576385; doi:10.3389/fgene.2021.728091)
Supplement: Supplementary file 4 [file DataSheet1.docx]

**Supplementary_Material S1. Demographic and clinical features of 8 case subjects**

|  | HC1 | HC2 | HC3 | IG1 | IG2 | AG1 | AG2 | AG3 |
| --- | --- | --- | --- | --- | --- | --- | --- | --- |
| Age(years) | 41 | 36 | 45 | 37 | 40 | 44 | 42 | 38 |
| Gender | Male | Male | Male | Male | Male | Male | Male | Male |
| Disease duration (months) | - | - | - | 31 | 33 | 25 | 2 | 12 |
| BMI（18.50~24.90kg/m2） | 23.41 | 22.86 | 23.55 | 24.16 | 24.05 | 24.62 | 25.16 | 24.23 |
| CRP（0.00~9.00mg/L） | 0.21 | 0.53 | 0.81 | 3.24 | 2.29 | 35.57 | 66.77 | 33.24 |
| ESR（0~21mm/h） | 2 | 3 | 2 | 6 | 10 | 30 | 49 | 123 |
| WBC（3.50~9.50×109/L） | 6.88 | 5.79 | 6.66 | 6.91 | 7.62 | 11.09 | 13.02 | 12.11 |
| GR（1.40~7.10×109/L） | 4.52 | 4.15 | 4.55 | 5.01 | 4.88 | 7.97 | 8.13 | 8.45 |
| LY（0.70~4.75×109/L） | 1.12 | 0.99 | 1.61 | 1.50 | 2.05 | 2.01 | 4.12 | 3.01 |
| Mo（0.10~0.60×109/L） | 0.34 | 0.43 | 0.22 | 0.35 | 0.61 | 0.78 | 0.75 | 0.58 |
| sUA（210.0~430.0μmol/L） | 366.1 | 380.9 | 378.7 | 440.6 | 450.6 | 567.9 | 699.8 | 487.4 |
| GLU (3.85~6.11mmol/L） | 3.99 | 5.41 | 5.39 | 5.77 | 5.86 | 4.81 | 5.88 | 4.26 |
| TG（0.50~1.80mmol/L） | 1.11 | 1.13 | 1.22 | 1.09 | 1.54 | 1.39 | 1.28 | 1.55 |
| TC（3.10~5.72mmol/L） | 4.33 | 3.87 | 3.85 | 4.38 | 4.76 | 4.88 | 4.02 | 4.67 |
| HDL (0.90~1.60mmol/L) | 1.21 | 1.23 | 1.33 | 1.23 | 1.45 | 1.42 | 1.28 | 1.36 |
| LDLC (1.90~3.40mmol/L) | 2.98 | 2.88 | 3.01 | 2.56 | 2.78 | 3.11 | 3.12 | 2.55 |
| VLDL (0.25~0.81mmol/L) | 0.45 | 0.55 | 0.77 | 0.69 | 0.78 | 0.74 | 0.79 | 0.71 |

HC: healthy controls ; IG: patients with intercritical gout; AG: patients with acute gout flare; BMI：Body Mass Index; CRP: reactive protein; ESR: erythrocyte sedimentation rate; WBC: white blood cell counts; GR: neutrophile granulocytecounts; LY: lymphocyte counts; Mo: monocyte counts; sUA：serum uric acid; GLU: serum glucose; TG: triglycerides; TC: Total Cholesterol; HDL: high-density lipoprotein; LDL: low-density lipoprotein; VLDL: very low-density lipoprotein.
